# A Brief Review on Chemoresistance; Targeting Cancer Stem Cells as an Alternative Approach

**DOI:** 10.3390/ijms24054487

**Published:** 2023-02-24

**Authors:** Belén Toledo, Aitor González-Titos, Pablo Hernández-Camarero, Macarena Perán

**Affiliations:** 1 Department of Health Sciences, University of Jaén, Campus de las Lagunillas, 23071 Jaen, Spain; 2 Excellence Research Unit “Modeling Nature” (MNat), University of Granada, 18016 Granada, Spain; 3 Biopathology and Regenerative Medicine, Institute (IBIMER), University of Granada, Centre for Biomedical Research (CIBM), 18071 Granada, Spain

**Keywords:** chemoresistance, DNA-damaging drugs, cancer stem cells, drug metabolism, *p53*, reactive oxygen species, drugs pumps, DNA repair, differentiation therapy

## Abstract

The acquisition of resistance to traditional chemotherapy and the chemoresistant metastatic relapse of minimal residual disease both play a key role in the treatment failure and poor prognosis of cancer. Understanding how cancer cells overcome chemotherapy-induced cell death is critical to improve patient survival rate. Here, we briefly describe the technical approach directed at obtaining chemoresistant cell lines and we will focus on the main defense mechanisms against common chemotherapy triggers by tumor cells. Such as, the alteration of drug influx/efflux, the enhancement of drug metabolic neutralization, the improvement of DNA-repair mechanisms, the inhibition of apoptosis-related cell death, and the role of *p53* and reactive oxygen species (ROS) levels in chemoresistance. Furthermore, we will focus on cancer stem cells (CSCs), the cell population that subsists after chemotherapy, increasing drug resistance by different processes such as epithelial-mesenchymal transition (EMT), an enhanced DNA repair machinery, and the capacity to avoid apoptosis mediated by BCL2 family proteins, such as BCL-XL, and the flexibility of their metabolism. Finally, we will review the latest approaches aimed at decreasing CSCs. Nevertheless, the development of long-term therapies to manage and control CSCs populations within the tumors is still necessary.

## 1. Introduction

**Figure 1:**
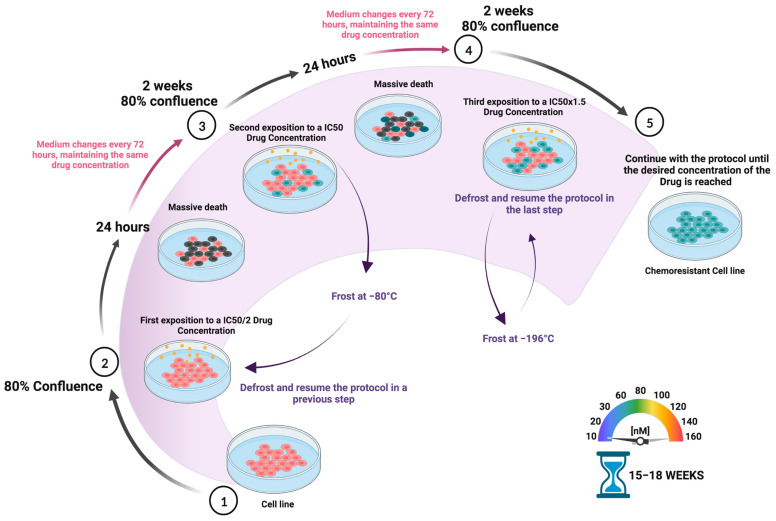
Schematic representation of the protocol used to develop drug-chemoresistant cell lines. To establish resistant tumor cell lines, increasing concentrations of the chemotherapeutical drug are periodically administered during a certain period of time, between 15 and 18 weeks, depending on the cell type. Briefly, cells are incubated with increasing concentrations of the drug in the culture medium from IC50/2 to the desired final concentration. The medium is changed every 72 h, while maintaining the drug concentration. Passage is done when cells acquire an 80% confluence (approx. every 2 weeks). It is recommended to freeze a cell stock in liquid nitrogen when acclimated to each drug concentration (the level of chemoresistance is not affected) as it is increased.

Currently, chemotherapy is still considered an irreplaceable front-line therapeutic strategy to combat almost all types of cancers, but multidrug resistance represents a common hurdle that deeply compromises clinical outcomes. Therefore, it is key to identify new resistance biomarkers and to analyze their predictive potential in order to guide treatment regimens [1]. Furthermore, a better understanding of the underlying drug tolerance mechanisms may be critical in achieving alternative therapies to improve oncological patients’ prognosis. However, it is challenging since a high heterogeneity of chemoresistance markers expression profiles has been observed between different tumors and even between different cell lines of the same tumor type [2]. In this regard, the development of drug-resistant cell lines is essential to study chemoresistance mechanisms. Figure 1 summarizes the procedure to obtain in vitro chemoresistant cell lines by mimicking the conditions experienced by cancer patients during chemotherapy. The use of increasing concentrations of chemotoxic agents represents a common experimental procedure with the aim of establishing stable, drug-tolerant tumor cell lines in vitro [3]. Although this method is very reliable and reproducible, some potential limitations should be kept in mind: first, since only a small fraction of the bulk population of cells will show chemoresistance, it is recommended to start with a large population of cells (a minimum of 10^6^ cells); second, it is very important to use the same freshly prepared drug stock for long-term use; third, it is crucial to start the protocol by determining the IC50 in each individual cell line, as variations may occur when using a new batch of cells (Figure 1). In general, it is crucial to choose a cell line that is resistant to chemotherapy with a relatively low referenced IC50 value for the drug of interest; finally, it is mandatory to avoid any contamination, including Mycoplasma, since it has been proved that microbiota can increase chemoresistance [4].

**Figure 2:**
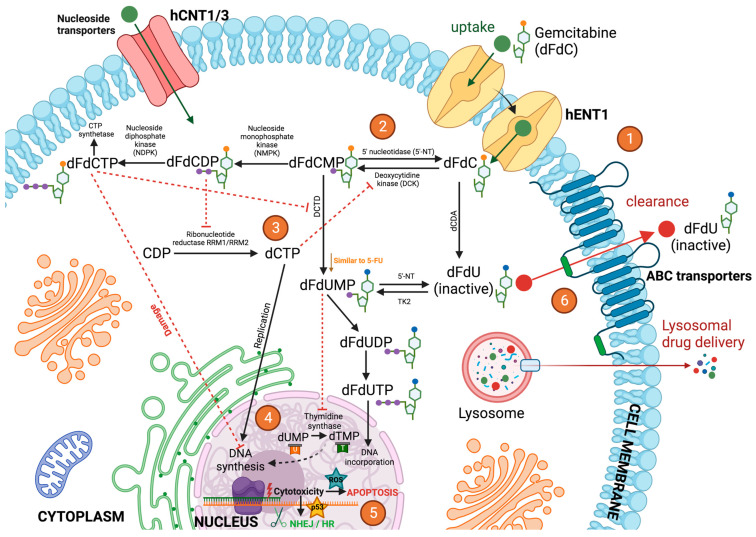
Mechanism of action of gemcitabine via cellular metabolism. (1) Once administered, gemcitabine (dFdC) is transported into cells by nucleoside transporters such as hENT1. (2) Gemcitabine is then phosphorylated into gemcitabine monophosphate (dFdCMP) by deoxycytidine kinase (DCK), and dFdCMP is subsequently phosphorylated to gemcitabine diphosphate (dFdCDP) and gemcitabine triphosphate (dFdCTP) by nucleoside monophosphate kinase (NMPK) and nucleoside diphosphate kinase (NDPK). (3) dFdCDP potently inhibits ribonucleotide reductase (RR) RRM1/RRM2, resulting in a decrease in the competing deoxyribonucleotide pools necessary for DNA synthesis. RR transforms cytidine diphosphate (CDP) into deoxycytidine diphosphate (dCDP), and its inhibitory effect leads to the decreased concentration of competitive deoxycytidine triphosphate (dCTP) pool cells required for DNA synthesis, thus promoting the binding of dFdCTP to DNA. Gemcitabine exerts its cytotoxic effect mainly through the inhibition of DNA synthesis by being incorporated into the DNA strand as the active dFdCTP. dFdCTP suppresses activation of dFdCMP by inhibiting deoxycytidine monophosphate deaminase (DCTD). (4) dFdCTP in the nuclei inhibits Deoxyribonucleic Acid (DNA) and Ribonucleic Acid (RNA) synthesis. 2′,2′-difluorodeoxyuridine monophosphate (dFdUMP) inhibits thymidine synthase (TS), resulting in the depletion of the deoxythymidine monophosphate (dTMP) pool, but it can also be phosphorylated to 2′,2′-difluorodeoxyuridine triphosphate (dFdUTP) for its DNA incorporation or dephosphorylated to 2′,2′-difluorodeoxyuridine (dFdU) and then transported out of the cells. (5) Gemcitabine cytotoxicity activates DNA damage responses such as NHEJ, HR, and *p53*. Gemcitabine can also induce DNA damage by further promoting the accumulation of ROS, thereby enhancing apoptosis. On the other hand, *p53* mutations can contribute to intracellular ROS accumulation and thus to genomic instability, which is characteristic of malignant cells. (6) The majority of dFdC is inactivated mainly by the deoxycytidine deaminase (dCDA)-mediated conversion to dFdU and then excreted through the ABC transporters. Deamination of dFdCMP to dFdUMP by DCTD and its subsequent dephosphorylation form, dFdU, is another inactivation pathway of dFdC.

Amongst the huge variety of anti-cancer agents, we have focused on DNA-damaging drugs (DDDs), gemcitabine (Gem) (dFdC), 5-fluorouracil (5-FU), cisplatin, and doxorubicin as the main treatments for high-incidence tumors. Gemcitabine and 5-FU are “anti-metabolites”, analogous to pyrimidine-based nucleotides, namely, cytosine and thymine/uracil, respectively, while cisplatin is an alkylating agent that generates DNA adducts and doxorubicin is a topoisomerase II inhibitor [5]. Tumor cells protect themselves from the aggression of cytotoxic agents through a series of mechanisms that range from preventing the entry of the drug to repairing the damage caused (Figure 2). Accordingly, in the face of aggression, tumor cells raise their first line of defense, preventing the entry of the drug and favoring its efflux through membrane pumps to compromise intracellular drug accumulation [6]. Further, DDDs are kept out of the nucleus where they perform their cytotoxic action by interfering mechanisms, for instance, lysosomal sequestration, as described for platinum-based treatment resistance [7]. In addition, the inactivation of chemotherapeutic agents, such as gemcitabine or 5-FU, has also been shown to contribute to the clinical failure of chemotherapy [8] (Figure 2).

Moreover, the basal DNA repair mechanism of eukaryotic cells is enhanced in tumor cells after prolonged exposition to cytotoxic agents, implying the resistance to treatment in many cancer types including diffuse large B-cell lymphoma [9]. Finally, apoptosis evasion, mainly through *p53* mutations, has been described in a wide spectrum of cancers as a mechanism of cytotoxic drug tolerance [10].

While highly proliferative cancerous cells are more sensitive to DDDs, low-proliferative cancer cells tend to be more resistant. In this sense, cancer stem-like cells (CSCs) have been defined as low-proliferative/quiescent cells, with high invasive, metastatic, and chemoresistant potentials, which undergo the epithelial-to-mesenchymal transition (EMT) process [11]. Thus, the acquisition of a CSC-like phenotype upon exposure to chemotherapy can be considered as a pro-survival tumor cells’ mechanism [12].

CSCs chemo and radio resistance are both supported by the intrinsic characteristics of their quiescent nature: (i) increased DNA repair mechanism, (ii) the ability to escape cell death [13], and (iii) the flexibility of their metabolism [14]. In addition, CSCs chemoresistance is also due to an increased drug efflux through ABC transporters [15]. In fact, a wide variety of studies have shown the strong relationship between stemness developed through upregulation of stemness markers, such as Nanog, OCT4, SOX2, and CD44, and drug resistance via increased drug efflux [16]. Similarly, it has been demonstrated that the dedifferentiation of melanoma cells toward a CSC-like status was accompanied by an increased xenobiotic efflux capacity and thus, an alteration of the therapeutic agent uptake [17]. Other studies have correlated EPCAMhigh/CD44+ colorectal CSCs with oxaliplatin tolerance through increased DNA repair capacity, altering the cell cycle checkpoints or ROS scavenging [18]. Notably, the authors also mentioned some molecular pathways, i.e., Notch, WNT/β-Catenin, and the Janus kinase/signal transducer and activator transcription (JAK/STAT), which are significantly involved in the CSCs maintenance and thus, in the acquisition of a chemoresistant phenotype. In the same context, Matou-Nasri and colleagues identified the key role of the p38/MAPK and NFKβ signaling pathways in the survival of acute myeloid leukemia CSCs and their chemoresistance to 5-fluorouridine through the inhibition of the therapy-related induction of apoptosis [19]. Specifically, the Janus kinase/Signal Transducers and Activators of Transcription (JAK/STAT3) pathway has been shown to play an important role in CSCs. This pathway is activated by interleukine-6 (IL-6) and the epithelial growth factor (EGF), among others factors [20]. Activated JAK/STAT3 triggers JAK activation, which phosphorylates STAT3. P-STAT3 dimerizes and enters the nucleus inducing the expression of genes related to cancer progression and epithelial to mesenchymal transition (EMT) and stemness, resulting in an increase in chemoresistance [20,21]. In addition, the JAK/STAT3 pathway in breast cancer has been shown to increase chemoresistance via the regulation of the lipid metabolism-activating fatty acid oxidation [22]. Other studies have shown that an overexpression of STAT3 in colorectal CSCs increased chemoresistance while STAT3 degradation enhanced cell chemosensitivity and decreased stem cell markers expression [23].

Here, we will compare tumor cells chemoresistance mechanisms against common treatments in high-incidence tumors. Furthermore, we will focus on cancer stem cells (CSCs) as key players in cancer drug-resistance, introducing novel approaches to reduce this cell population and therefore, chemoresistance.

## 2. Boarding and Landing Gates

To exert their anti-cancer effects, DDDs have to reach their molecular targets inside the nucleus, mainly DNA and/or DNA synthesis-related enzymes. Undoubtedly, the trafficking across the plasma membrane and the drug influx/efflux ratio determine the cytotoxic agent intracellular concentration. Thus, dysfunctions in drug uptake pumps lead to an increase in chemoresistance. For instance, Zeng and co-workers (2021) described the volume-regulated anion channel (VRAC) as a mediator of cisplatin uptake. Moreover, it has been documented that the cisplatin influx promoted by the organic cation transport 1 (OCT1) in esophageal squamous cell carcinoma shows a significant positive correlation between a low expression of OCT1 and a reduced sensitivity to cisplatin, along with a poor prognosis [24].

In addition, the ion transporters OCT1, OCT2, OCT3, and OATP1A2 (organic anionic transporter 1A2) have been shown to promote the cellular uptake of doxorubicin under physiological conditions [25]. Furthermore, Wang and colleagues reported the participation of the OAT2 transporter in 5-FU uptake in hepatocellular carcinoma cells, which explained the correlation between OAT2 downregulation and acquired chemoresistance [26].

On the other hand, the increased efflux of therapeutic agents has been widely described as a central mechanism leading to multidrug resistance, with special emphasis on the upregulation of the ATP-binding cassette (ABC) superfamily with up to 48 different subtypes [27]. The huge heterogeneity among efflux pumps has clinical relevance since it dictates the substrate-binding affinity. In fact, Mora Lagares and colleagues (2021) [28] have shown the implication of ABC transporters transmembrane domains, with a large proportion of non-conserved residues, in the establishment of the substrate-binding pocket, suggesting different substrate specificities that are characteristic of each ABC member. According to this idea, a specific cytotoxic drug may be recognized and expelled out of the tumor cell by only certain ABC pumps. For instance, gemcitabine is ejected from the cells by overexpressed ABCC4 [29] or ABCC5 [30] transporters, leading to chemoresistance in pancreatic cancer treatments. It has also been demonstrated that the downregulation of hENT1, a nucleotide transporter associated with gemcitabine uptake, may also contribute to the acquisition of chemoresistance by pancreatic cancer cells [31] (Figure 2). In regard to cisplatin, some studies identified ABCB1 (MDR1), ABCG2 (BCRp), ABCC1, and ABCC4 as mediators of drug extrusion, thereby contributing to cisplatin tolerance [32]. Similarly, ABCA5, ABCC2, and ABCB5 [33] have also been reported to exert the same effect.

Additionally, 5-FU has been documented in colorectal cancer cells to be expelled from the intracellular space by ABCC11, but surprisingly, not by the widely recognized ABCB1 drug efflux pump [34]. In a recent study using non-small lung carcinoma cells, it was reported that 5-FU can also be a substrate of ABCA5 and ABCC1 transporters [33]. Moreover, in a hepatocellular carcinoma study, the overexpression of ABCB1, ABCB5, ABCC1, or ABCG2 had the potential to induce resistance to doxorubicin, and this fact was prevalent in hepatic CSCs rather than in non-stem cancerous cells [26].

Furthermore, the subcellular distribution of chemotherapeutic agents is key to supporting their cytotoxic effect. In this regard, it has been shown that the major vault protein (MVP) can promote doxorubicin transference from the nucleus to the cytoplasm and then to the extracellular space, inducing drug tolerance [35,36]. Reinforcing the importance of subcellular drug location, the copper transporter ATP7B has been shown to induce platinum-derived compounds lysosomal sequestration and their subsequent exocytosis, thereby promoting cisplatin chemoresistance in ovarian cancer cells [7].

Collectively, these data reflect the importance of the bidirectional trafficking across the plasma membrane, which is mediated by multiple transporters. Nevertheless, it is relevant to note that different tissues, tumor types, and even distinct cell lines of the same tumor may exhibit specific expression patterns of such transporters [25].

## 3. Drug Metabolism

Antimetabolites cytotoxic drugs exert their effect by incorporation into RNA and DNA molecules producing fatal errors, which lead to cell death. Gemcitabine and 5-FU are representative examples of antimetabolites that are analogous to pyrimidine-based nucleotides, namely cytosine and uracil/thymine, respectively [37] (Figure 2). Thus, the pyrimidine metabolic activity of target tumor cells may significantly influence drug availability and cytotoxic efficacy (Figure 2). Of note, dihydropyrimidine dehydrogenase (DPD) has been shown to be an important enzyme in the pyrimidine catabolic route which catalyzes a two-electron reduction in pyrimidine bases [38]. In fact, DPD deficiency in bladder cancer cells has been associated with gemcitabine sensitivity, whereas the overexpression of *DPYD* (DPD-coding gene) was linked to gemcitabine resistance via its catalytic inactivation [8] (Figure 2). Similarly, *DPD* upregulation in colorectal cancer has also been shown to promote 5-FU tolerance through intracellular 5-FU transformation into inactive metabolites [39].

Moreover, cytidine-metabolizing enzymes, such as cytidine deaminases (CDA), have been shown to interfere with gemcitabine-based therapies. A representative example is CDA, whose upregulation has been related to gemcitabine resistance in pancreatic cancer via metabolic neutralization [1]. Interestingly, other cytidine deaminases, such as the AID and APOBEC family, have also been related to key roles in cancer biology [40]. Hence, it seems reasonable to propose a relevant crosslink between gemcitabine efficiency and these enzymes. In the case of CDA, its gemcitabine-neutralizing capacity has also been detected outside tumor cells, for instance in systemic blood circulation [12]. Parallelly, the secretion of pyrimidine nucleosides, such as deoxypyrimidine dehydrogenase (*DPD*), by tumor-associated macrophages (TAMs) in pancreatic ductal adenocarcinoma has been observed. Deoxypyrimidine dehydrogenase promotes gemcitabine resistance through the competitive attenuation of its uptake by tumor cells and by increasing its metabolic neutralization [41]. Furthermore, hypoxic TAMs have been proven to be the main contributors to *DPD* expression in colorectal cancer, implying the relevance of surrounding stromal cells in promoting resistance to chemotherapeutic antimetabolites [42]. Importantly, Malier and colleagues also noted that mice-derived macrophages did not express significant levels of *DPD*, contrary to human TAMs.

Once inside the cells, gemcitabine activated metabolites: difluorodeoxycytidine monophosphate (DFDCMP), the diphosphate form (DFDCDP), and subsequently, the triphosphate compound (DFDCTP) are phosphorylated by the enzyme deoxycytidine kinase (dCK) before achieving therapeutic effectiveness [43,44] (Figure 2). Concordantly, it has been revealed that an increased expression of dCK in high-grade meningioma cells determined the intracellular activation of gemcitabine leading to a significant increase in drug sensitivity [45]. Along with its misincorporation into synthesizing DNA strands (DFDCTP metabolite), gemcitabine (DFDCDP form) can also hamper the synthesis and repair of DNA via the inhibition of ribonucleotide reductase (RR). Indeed, the high expression of RR and the activation of the RR large subunit (RRM1) were associated with poorer patient outcomes and gemcitabine resistance in pancreatic cancer [43] (Figure 2).

On the other hand, 5-FU has to be converted to the active metabolite: fluorodeoxyuridine monophosphate (FDUMP) by mediation of enzymes such as thymidine kinase 1 (TK1) in order to exert its cytotoxic effect [46]. 5-FU causes DNA damage by being mistakenly incorporated into DNA- and RNA-based molecules, and by the induction of thymidine synthase (TS) inhibition, thus, hampering de novo thymidine synthesis. Moreover, the upregulation of TS has been shown to attenuate 5-FU cytotoxicity and to lead to drug resistance in a cholangiocarcinoma study [47].

Regarding CSCs, many studies have reported that the acquisition of a stem-like phenotype by cancerous cells may be accompanied by a deep metabolic reprogramming. To note, glycolysis is commonly enhanced in CSCs, as has been described in the case of glioblastoma CSCs [48]. The authors specified that this phenomenon along with the acquisition of a stem-like phenotype were induced by the long non-coding RNA HULC. In a similar way, another study has exposed that biomechanical forces derived from the extracellular matrix contributed to the dedifferentiation of colorectal cancer cells toward CSCs through the enhancement of glycolysis and HIF1 expression [49]. However, it has been highlighted that the metabolic reprogramming of CSCs may be much more flexible/reversible according to their phenotypic plasticity. Indeed, CSCs are able to transit between a quiescent, low-metabolic phenotype with little energy needs and a proliferative behavior with high energy costs [50]. Interestingly, it has been revealed that the acquisition of cisplatin tolerance by non-small cell lung carcinoma cells was associated with their glycolysis/oxidative phosphorylation metabolic flexibility and with an increased mitochondrial function [51]. Moreover, the dedifferentiation of cancerous cells toward CSCs can also be accompanied by other metabolic alterations. In regard to this, the overexpression of OCT4 has been related to the CSC-like phenotype along with the enhancement of both glycolysis and the oxidative pentose phosphate pathway [52]. These events may be of special relevance considering the well-known role of the pentose phosphate pathway in the defense against reactive oxygen species (ROS), which can also be correlated with the acquisition of chemoresistance (see the following section). According to the many metabolic changes surrounding the CSC-like phenotype, it seems reasonable to hypothesize that nucleotide metabolism could also be altered in CSCs. In agreement, it has been confirmed that the TS enzyme may be essential for the maintenance of the CSC-like status of triple-negative breast cancer cells, which has also remarkably been associated with an increased activity of DPD enzyme [53]. In addition, others observed that the overexpression of RRM2 (related to nucleotide synthesis) was closely correlated with the stemness of squamous cells of oral carcinoma [54]. These facts may be relevant considering the participation of DPD, RRM2, and TS enzymes in the drug tolerance of cancerous cells, as mentioned above.

## 4. DNA Damage Repair and Cell Proliferation

Upon reaching the nucleus, DDDs cause a variety of DNA lesions. Depending on the level of DNA damage, several types of DNA-damage responses (DDRs) are triggered and these can be classified into two main groups: (i) pro-survival responses (i.e., DNA repair and/or cell cycle arrest/premature cell senescence) and (ii) cell death (i.e., pro-apoptotic signaling). Specifically, DDRs are characterized by complex and multifactorial phosphorylation cascades comprising a wide variety of molecules such as DNA sensors (i.e., MRN complex, ATM, or ATR), DDR transducers such as checkpoint kinases (for example CHK1 or CHK2), and mediators/effectors such as *p53* and executioners (i.e., DNA repair-, cell cycle arrest-, or apoptosis-related factors) [55]. Considering that the final goal of cancerous cells relies on surviving at all costs, the reported association between the optimization of DDR through *ATM/ATR/p53* axis upregulation and the tolerance to chemotherapeutic drugs such as gemcitabine in pancreatic cancer is not surprising at all [56]. Relevantly, some degree of specificity in the induction of certain DDR pathways according to the type of DNA damage has been shown. For instance, the activation of ATM/CHK2 signaling has been mainly correlated to double-stranded DNA breaks while the enhancement of the ATR/CHK1 axis has been mainly linked to single-stranded DNA breaks [57,58]. Similarly, different DNA repair mechanisms have been described according to the type of DNA damage. For instance, in the repair of double-strand DNA breaks, the high-fidelity homologous recombination (HR) or the error-prone non-homologous end-joining (NHEJ) are involved [59] (Figure 2). On the other hand, nucleotide excision repair (NER) is induced by DNA adducts [60] and single-stranded DNA breaks are repaired by base-excision repair (BER) [61]. Generally, DDDs can trigger several DNA repair responses, as has been revealed in the case of gemcitabine-based treatment, which can induce either NHEJ [62] or HR [63]. Nonetheless, some trends with certain chemotherapeutic treatments have been identified, such as NER activation to overcome DNA adducts caused by alkylating platinum compounds [64]. However, HR repair machinery has also been shown to be involved in the resolution of DNA damage induced by a wide spectrum of cytotoxic agents, including gemcitabine, 5-FU, cisplatin, and doxorubicin [5]. The development of suitable experimental protocols to simultaneously test a broad range of DNA repair responses may be of great interest. In regard to this, Ge and co-workers pointed out that the transcriptional profiling of DNA repair-associated genes does not always correlate with the real DNA repair capacity. Therefore, they developed a “cometchip platform” based on the previously established comet assay that is suitable for the parallel assessment of multiple repair pathways such as BER, NER, and NHEJ [61]. Furthermore, it may be relevant to note that DNA damage and, thus, the DNA repair capacity can also be enhanced in some cellular contexts that are different from the exposure to therapeutic compounds. For instance, a high-proliferative phenotype may elicit excessive proliferative stress, which has been associated with the overactivation of DNA repair signaling [65]. On the other hand, a close correlation between chronic inflammation and the induction of oxidative stress and DNA damage has been established [66]. Considering the uncontrolled proliferation of tumor cells and the documented pro-inflammatory microenvironment surrounding solid tumors since the earliest stages of their growth [67], a remarkable DNA repair capacity even in the absence of antitumor drugs seems like a reasonable suggestion. Importantly, the reinforcement of DDRs by a pro-inflammatory microenvironment may be a significant difference between in vitro and in vivo chemoresistance-based studies.

Parallelly, cell cycle arrest promoted by DNA damage is a well-known cellular response in physiological conditions [68]. In fact, DNA-induced cell cycle arrest after chemotherapy might be a pro-survival cellular response, which may lead to chemoresistance by providing sufficient time to complete DNA repair. However, some studies have interpreted the inhibition of DNA damage-mediated cell cycle arrest by a cancerous cell as a drug-resistant mechanism [69]. With regard to this controversy, the authors also noted the importance of carefully considering the balance between the DNA repair potential and the cell cycle arrest/growth inhibition in chemoresistance-based research. Moreover, distinguishing between punctual/short-term cell cycle arrest and permanent/long-term cell cycle blockade (also known as “cellular senescence”), both promoted by DNA damage, may be relevant to clarify such a dichotomy. In this sense, the induction of cellular senescence in several types of cancers upon treatment with doxorubicin and etoposide has been shown [70]. On one hand, the induction of tumor senescence as an anti-cancer strategy to inhibit tumor proliferation and growth has been considered [71]. Nevertheless, the plasticity and reversibility of the senescent-like phenotype leading to a more aggressive and invasive behavior and even the promotion of disease relapse and metastasis have also been pointed out [70]. Interestingly, these are well-documented negative events that are closely related to the CSC population, the EMT process, and chemoresistance [72]. In agreement, the promotion of cellular senescence and a stem-like phenotype after sustained and long-term DNA-damaging conditions, i.e., radiotherapy, has been identified [73]. Additionally, it has been indicated that DDDs can also be associated with the EMT process and tumorogenesis [74]. Therefore, cellular senescence and the acquisition of a CSC-like phenotype seem to support chemotherapy through pro-survival tumor cell behavior [12].

## 5. *p53* and Reactive Oxygen Species (ROS) Levels: Role in Chemoresistance

Nuclear DNA damage triggers cell death mainly by the activation of *p53*, also known as “the guardian of the genome” [75]. In fact, *p53* phosphorylation leads to the phosphorylation cascade toward apoptosis-based cell death [55]. In healthy cells, the expression of *p53* is usually low, with a half-life of about 20 min [76]. However, after cellular stress, the *p53* half-life extends to several hours, promoting different responses such as cell-cycle arrest, senescence, apoptosis, regulation of cellular energy metabolism, antioxidant defense, DNA repair, and immune system regulation [77]. Accordingly, the mutant *p53* protein has been shown to interfere with a variety of processes such as the regulation of cell survival, DNA damage repair, and drug resistance [78]. *p53* versatility has been shown to be driven by different levels of phosphorylation and it has been shown that *p53* can act as a “cell cycle arrestor” permitting DNA repair and cell survival when it is phosphorylated on Serine15 (Ser15) and/or Serine20 (Ser20). Nevertheless, additional phosphorylation on Ser46 under severe DNA damage conditions may change its role to “killer”, leading to apoptosis [55]. In summary, as a cellular “gatekeeper”, *p53* recognizes whether DNA damage is irrevocable and acts accordingly by inducing apoptosis [77,79]. Therefore, *p53* plays a dual role by activating either a mechanism that leads to apoptosis or one that enhances DNA repair and cell survival.

The mutated *p53* gene (TP53) has been detected in approximately 50% of all human tumors, such as breast, brain, lung, or colorectal carcinomas, among others [80], which may deeply condition the prognosis and clinical outcomes of oncological patients. Particularly, germline *p53* mutants are mainly associated with Li–Fraumeni Syndrome, which is characterized by a high risk of oncogenesis [80,81]. Some mutations can make the *p53* protein unable to recognize and interact with *p53*-binding sites located in its target genes. In accordance with this, Donzelli and co-workers (2012) showed that mutant *p53* conferred tolerance to doxorubicin, cisplatin and 5-FU by procaspase-3 downregulation, and apoptosis inhibition [82].

Furthermore, *p53* mutations promote the acquisition of new and distinct oncogenic properties by interacting with different genes, which is generally referred to as “gain of function” alterations (GOF) [83]. Specifically, mutant *p53* can reach the promoter of target genes through the interaction with several sequence-specific transcription factors including NF-Y, E2F1, NF-kB, and the Vitamin D receptor (VDR) [84]. GOF mutations may promote tumor progression and possibly lead to resistance to a variety of anticancer drugs. For instance, studies have shown that mutant *p53* may be associated with chemoresistance in an independent way of its pro-apoptotic role, increasing the expression of the MDR1 efflux pump (ABCB1) [85]. Additionally, a relationship between mutant *p53* and EFNB2 (ephrin-B2), a receptor tyrosine kinase involved in cell invasion, migration, angiogenesis, and tumor resistance, has been established [86]. Moreover, it was reported that mutant *p53* increases EFNB2 expression in colorectal carcinoma cell lines upon treatment with 5-FU [87]. The authors also showed that EFNB2 induced 5-FU resistance through the upregulation of the ABCG2 drug efflux transporter, mediated by the activation of the c-Jun/JNK signaling pathway. Further, it has been observed that *p53* knockdown reduced cell proliferation and resistance to cisplatin, adriamycin, and etoposide in several cancer cell lines [88]. On the contrary, an overexpression of *p53* has been associated with gemcitabine tolerance in pancreatic cancer [89]. Thus, *p53* phosphorylation status and not only its expression patterns should be taken into account to explain observed controversies regarding *p53* expression and chemoresistance.

Apart from the high rate of *TP53* mutations, the remaining 50% of cancers may exhibit *p53* dysregulation or alterations in *p53*-related pathways [90]. To note, it has recently been discovered that the overexpression of CD147 may promote the acquisition of gemcitabine resistance by pancreatic tumor cells by interfering with the activation of *p53* upon *ATM/ATR/p53* complex formation, thus preventing cell apoptosis (REF: CD147). Similarly, the overexpression of MSIM2 may cause resistance to gemcitabine and cisplatin in pancreatic cancer by negatively regulating *p53* (REF: MSIM2). Notably, *p53* has been documented to control its protein levels through a negative feedback loop involving MDM2 [91]. *P53* can act as a transcription factor activating MDM2, which can then enhance *p53* protein ubiquitination and degradation [92]. Upon cellular stress, mediated for example by DNA damage, MDM2 activity decreases, leading to an increase in *p53* levels through its protein stabilization (non-degradation) [93]. As a consequence, a re-increase in MDM2 is observed, which in turn, may promote *p53* protein degradation. In physiological conditions, nuclear concentrations of both *p53* and MDM2 may be mutually maintained at low levels [91]. Nonetheless, the dysregulation of *MDM2/p53* balance could be associated with severe disorders such as tumorigenesis and poor clinical prognosis [93], hence, interfering with such a feedback control may represent a promising therapeutic strategy in oncology. In addition, *p53* dysregulation could also lead to deficient immune system responses [94], which may play a key role in cancer immune evasion. For instance, Major Histocompatibility Complex Class I (MHC-I) was described to be positively regulated by *p53*, promoting damaged cell recognition by T cells.

Parallelly, reactive oxygen species (ROS) levels have been reported to be increased in cancerous cells due to both environmental (smoking or UV) and internal mechanisms (ROS are considered as an inevitable by-product of cellular metabolism). Therefore, the increased metabolism of high-proliferative cancerous cells may result in elevated ROS production [95]. Furthermore, ROS generation can be related to well-known cancer features, for instance, the overactivation of oncogenes such as C-myc, Kras, or BRCA1, or the alteration of integrins during metastasis. In cancer cells, ROS could play a dual role: under basal conditions, ROS play a critical role in maintaining cellular proliferation and homeostasis [96], whereas high ROS concentrations may inhibit cell cycle progression and induce apoptosis [97] (Figure 2). Relevantly, many anticancer compounds, namely doxorubicin, cisplatin, or 5-FU, can induce DNA damage by further promoting the accumulation of ROS and thereby enhancing apoptosis [58]. Importantly, this study also indicated that ROS, like H_2_O_2_, can trigger the activation/phosphorylation of *p53* through a DDR-independent manner. Strikingly, mutant *p53* can also induce ROS accumulation due to the loss of its antioxidant potential, i.e., by causing antioxidant enzyme imbalance [98]. Hence, *p53* mutations can contribute, at least in part, to the intracellular ROS accumulation and thus, to the genomic instability characteristics of malignant cells [99]. As it has been revealed that cancer cells could enhance their antioxidant mechanisms to counterbalance excessive oxidative stress, this could be considered as chemoresistant behavior. For instance, the overexpression of antioxidant enzyme Isocitrate Dehydrogenase 1 (IDH1) reduced ROS levels and promoted gemcitabine resistance in pancreatic ductal adenocarcinoma [100]. Similarly, cisplatin can interact with endogenous nucleophiles such as reduced glutathione (GSH), which makes the redox balance prone to oxidative stress [101]. Thus, the observed susceptibility of cisplatin-based treatments to cytoprotective antioxidant molecules [102,103] may be determined in a double way: ROS direct neutralization and cisplatin activity mitigation by the overexpression of GSH. Several resistant routes can be triggered in response to elevated ROS levels, involving endoplasmic reticulum (ER) stress, autophagy, cell cycle perturbations, and the acquisition of a CSC-like phenotype through the EMT process [104]. Supporting the malignant cells’ adaptation to high ROS accumulation, recent evidence suggests that prolonged chemotherapy can reduce the overall ROS concentration, thus leading to drug tolerance [105]. As an additional example, gemcitabine-induced oxidative stress accompanied by an increase in antioxidant genes, i.e., *NRF2*, *SOD1*, *SOD2*, *CAT*, or *GPX1*, has been demonstrated [106]. Additionally, oxygen availability may significantly determine anticancer drug efficiency. While hypoxic conditions may limit ROS production, the recovery of normal oxygen levels can elevate the cytotoxicity of DDDs, such as doxorubicin, by increasing the generation of ROS and ultimately, alleviating the chemoresistance [107]. Therefore, the development of hypoxic environments in almost all solid malignancies may represent an important difference between in vitro and in vivo and/or clinical studies. However, the therapeutic feasibility of pro-oxidant drugs in cancer with mutant *p53* status remains to be well-defined [108].

## 6. Chemoresistance: Targeting CSCs as an Alternative Approach

**Figure 3:**
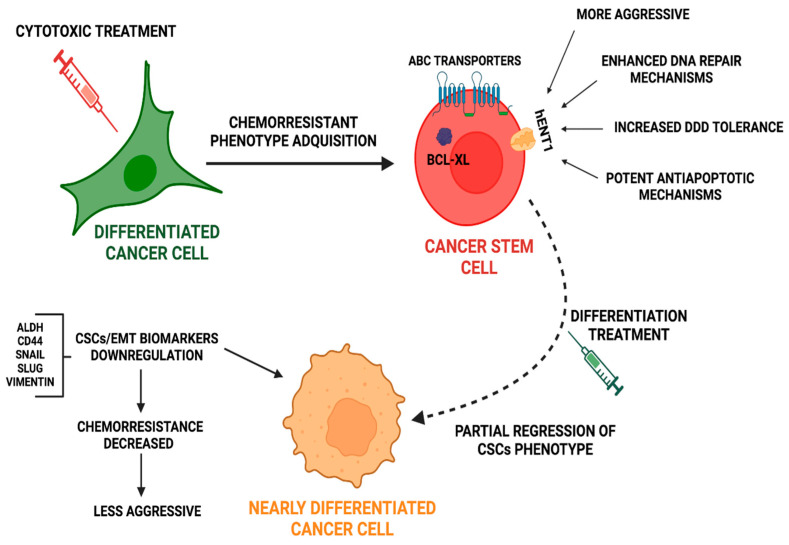
Cytotoxic treatments induce cancer stem cells phenotype acquisition together with increased chemoresistance. Approaches to re-differentiate CSCs could improve current treatment. After cytotoxic treatment, differentiated cancer cells acquire a quiescent CSCs-like phenotype, through the successful repair of damaged DNA. CSCs, after cytotoxic assault trigger protective mechanisms, such as the expression of ABC transporters and mechanisms to repair DNA, to increase tolerance to DDDs and to avoid apoptosis. Novel strategies focusing on new non-toxic anticancer compounds could induce regression of the CSC phenotype toward a differentiated phenotype with less chemoresistant potential.

As revised in previous sections, the acquisition of a quiescent, CSC-like phenotype by well-differentiated cancerous cells provides them with sufficient time for successful DNA repair after the cytotoxic assault. Nevertheless, CSCs chemoresistance can rely on multiple additional mechanisms. Indeed, the correlation between breast, colorectal, and lung CSCs, the upregulation of ABC family members, such as ABCC1, and the cisplatin and doxorubicin tolerance have been demonstrated [109] (Figure 3). Moreover, it has been revealed in a colorectal cancer study using 5-FU as a chemotherapeutic agent that a high drug metabolic detoxification capacity is a typical property of dormant CSCs [110]. In addition, CSCs usually exhibit enhanced DNA repair mechanisms in a similar way to healthy stem cells, which may lead to increased DDDs tolerance (Figure 3). What is more, the key importance of DNA repair-related pathways/proteins in the maintenance of a CSC-like phenotype, for instance, by regulating the EMT process, has been reported [111]. The chemoresistance capacity of CSCs also relies on avoiding apoptosis mediated by the enhancement of BCL2 family proteins, such as BCL-XL, as has been determined by the Medema group in a colorectal cancer study [112]. Interestingly, they documented the reliance of different antiapoptotic proteins according to the tumor progression stage, which may suggest the existence of distinct antiapoptotic mechanisms between healthy cells, well-differentiated cancerous cells, and CSCs. Altogether, these data reinforce the crucial importance of developing therapeutic strategies with the aim of eliminating such a cancer cell subpopulation (Figure 3).

In a tumor mass and surroundings, the CSCs population is maintained by the dedifferentiation of tumors cells located in the borders (revised in Hernández-Camarero et al., 2018) [113]. Thus, CSCs are a population that subsists after chemotherapy, enhance drug resistance, and reappear constantly upon tumor cell dedifferentiation. All these facts make the development of long-term therapies to manage and control the revival of CSCs populations necessary, while avoiding chemotherapy that is associated severe side effects (Figure 3).

In this regard, strategies that control and deregulate the CSCs population are interesting approaches.

For instance, targeting pathways that regulate CSCs has been proposed. One of the main candidates has been the Notch pathway and to this end, different strategies have been tested: (i) gamma-secretase inhibitors (GSIs) [114]; (ii) monoclonal antibodies targeting Notch signaling [115], and (iii) pan-Notch inhibition [116]. Another strong candidate has been the Wnt pathway, whose inhibition has been achieved by an IgG4 mAb (DKN-01) that targets Dkk1 and suppresses canonical Wnt signaling via negative feedback [117]. In addition, efforts have been undertaken to control the hedgehog signaling pathway, with the use of SMO inhibitors and GLI inhibitors [118]. Furthermore, CSC-directed immunotherapy has also received attention. CSC lysates are used to generate CSC-specific T cells that directly target the subpopulation of CSCs within tumors [119]. The relevance of these approaches is supported by their translation to the clinical arena. In fact, several clinical trials on therapies targeting CSCs are actually ongoing or have already been completed [120].

Nevertheless, the depletion of CSCs by itself has not been shown to be effective in reducing chemoresistance because other factors, such as the tumor microenvironment (TME) [121], play a key role in CSC regulation and stem maintenance via the transition of non-stem cells to stem cell states [122]. Thus, strategies should focus on directing CSCs differentiation (Figure 3) instead of CSCs eradication.

In conclusion, the development of novel therapeutic approaches directed against CSCs and the TME could reduce chemoresistance and the associated adverse effects.

## Data Availability

Not applicable.

## Abbreviations

5-FU	5-Fluorouracil
ABC	ATP-Binding Cassette
BER	Base-Excision Repair
CDA	Cytidine Deaminase
CDP	Cytidine Diphosphate
CDTD	Deoxycytidine Monophosphate Deaminase
CSCs	Cancer Stem-Like Cells
dCDA	Deoxycytidine Deaminase
dCDP	Deoxycytidine Diphosphate
dCK	Deoxycytidine Kinase
dCTP	Deoxycytidine Triphosphate
DDDs	DNA-Damaging Drugs
DDRs	DNA-Damage Responses
dFdC	Gemcitabine
dFdCDP	Gemcitabine Diphosphate
dFdCMP	Gemcitabine Monophosphate
dFdCTP	Gemcitabine Triphosphate
dFdU	2′,2′-Difluorodeoxyuridine
dFdUMP	2′,2′-Difluorodeoxyuridine Monophosphate
dFdUTP	2′,2′-Difluorodeoxyuridine Triphosphate
DFDCDP	Difluorodeoxycytidine Diphosphate
DFDCMP	Difluorodeoxycytidine Monophosphate
DFDCTP	Difluorodeoxycytidine Triphosphate
DNA	Deoxyribonucleic Acid
DPD	Dihydropyrimidine Dehydrogenase
dTMP	Deoxythymidine Monophosphate
EMT	Epithelial-to-Mesenchymal Transition
ER	Endoplasmic Reticulum
FDUMP	Fluorodeoxyuridine Monophosphate
Gem	Gemcitabine
GOF	Gain Of Functions
GSIs	Gamma-Secretase Inhibitors
GSH	Glutathione
HR	Homologous Recombination
IC50	Half-Maximal Inhibitory Concentration
IDH1	Isocitrate Dehydrogenase 1
JAK/STAT3	Janus kinase/Signal Transducers and Activators of Transcription
MHC	Major Histocompatibility Complex Class
MVP	Major Vault Protein
NDPK	Nucleoside Diphosphate Kinase
NER	Nucleotide Excision Repair
NHEJ	Non-Homologous End-Joining
NMPK	Nucleoside Monophosphate Kinase
OATP	Organic Anionic Transporter
OCT	Organic Cation Transport
ROS	Reactive Oxygen Species
RNA	Ribonucleic Acid
RR	Ribonucleotide Reductase
Ser	Serine
TAMs	Tumor-Associated Macrophages
TME	Tumor Microenvironment
TK1	Thymidine Kinase 1
TS	Thymidine Synthase
VDR	Vitamin D Receptor
VRAC	Volume-Regulated Anion Channel
